# Mesenchymal stem cells-regulated Treg cells suppress colitis-associated colorectal cancer

**DOI:** 10.1186/s13287-015-0055-8

**Published:** 2015-04-13

**Authors:** Rui-jing Tang, Su-nan Shen, Xiao-yin Zhao, Yun-zhong Nie, Yu-jun Xu, Jing Ren, Ming-ming Lv, Ya-yi Hou, Ting-ting Wang

**Affiliations:** The State Key Laboratory of Pharmaceutical Biotechnology, Division of Immunology, Medical School, Nanjing University, Nanjing, 210093 China; Jiangsu Key Laboratory of Molecular Medicine, Nanjing, 210093 China

## Abstract

**Introduction:**

Previous studies have produced controversial results regarding whether mesenchymal stem cells (MSCs) promote or inhibit tumor development. Given the dual role of MSCs in inflammation and cancer, in this study the colitis-associated colorectal cancer (CAC) model was used to examine whether umbilical cord tissue-derived MSCs could prevent neoplasm by inhibiting chronic inflammation.

**Methods:**

MSCs were obtained and identified using flow cytometry. Colitis-associated colorectal cancer model was induced using azoxymethane (AOM) and dextran sulfate sodium (DSS) and MSCs were injected intravenously twice. Levels of immune cells in mesenteric lymph node including regulatory T (Treg) cells were detected using flow cytometry. Naïve T cells and Jurkat cells were co-cultured with MSCs and the effect of MSCs on Treg cells differentiation was evaluated.

**Results:**

After injection through tail vein, MSCs could migrate to colon and suppress colitis-related neoplasm. This tumor suppressive effect was characterized by longer colon length, decreased tumor numbers and decreased expression of Ki-67. Moreover, MSCs alleviated the pathology of inflammation in the colitis stage of CAC model and inhibited inflammation cytokines both in colon and serum. Furthermore, Treg cells were accumulated in mesenteric lymph node of MSCs-treated mice while the percentage of T helper cells 2 (Th2) and Th17 were not changed. Of note, MSCs secreted transforming growth factor-β (TGF-β) enhanced the induction of Treg cells from naïve T cells. The conditioned medium of MSCs also activated Smad2 signaling, which has been reported to regulate Treg cells.

**Conclusions:**

These results proved that MSCs could migrate to colon tissues and induce the differentiation of Treg cells via Smad2 as so to inhibit the colitis and suppress the development of CAC.

## Introduction

The connection between inflammation and tumor development was noticed after Virchow demonstrated that cancer tended to occur at a site of chronic inflammation [1]. Colorectal cancer which includes hereditary, sporadic and colitis-associated colorectal cancer (CAC) is one of the most common malignancies. More and more evidence shows that chronic inflammation of the colon is an important factor for the progression of colorectal cancer [2]. Patients with inflammatory bowel disease (IBD), such as Crohn’s disease and ulcerative colitis, have a higher risk of colorectal cancer development than the healthy population. It is now becoming clear that tumor microenvironment, which is largely orchestrated by inflammatory cells, is an indispensable participant in the neoplastic process, including cancer cell proliferation, survival and migration [3]. These insights are fostering new anti-inflammatory therapeutic approaches to cancer [4].

Mesenchymal stem cells (MSCs), which are derived from a variety of tissues and have a fibroblast-like morphology, have the capability of self-renewal and differentiation. MSCs can migrate to the site of tissue damage induced by inflammation and play an anti-inflammatory role through regulation of the function of dendritic cells, natural killer cells (NK cells), T cells, and B cells [5]. MSC can also induce regulatory T (Treg) cells and maintain the capability of Treg cells [6-8]. These properties, which are useful for therapeutic purposes, have recently been found to be abused by cancer cells for their own end. In contrast, reports show that MSCs can inhibit tumor growth under certain circumstances. Our previous study has demonstrated that MSCs can alleviate inflammatory disorders in dextran sulfate sodium (DSS)-induced colitis [9]. Given the dual role of MSCs in inflammation and cancers, we hypothesized that MSCs may have an effect on the initiation and progression of CAC.

The role of the immune response in the formation of CAC is complicated. Chronic colitis accompanied by a large accumulation of T helper cell 1 (Th1), Th2 and Th17 promotes neoplastic risk, whereas excessive immunosuppression regulated by Treg cells enhances the survival of tumor cells [1,10,11]. Many researchers have reported that excessive Th1 cells in intestinal mucosa are the main reason for chronic colitis; these cells produce interferon (IFN)-γ and interleukin-2 (IL-2) [12,13]. Meanwhile, CAC was also characterized as a Th2/Th17 disease accompanied by overproduction of cytokines such as IL-4, IL-5, IL-13 and IL-17 [14,15]. Importantly, Treg cells, which are important in regulating immune responses by selectively suppressing effector T cells, are believed to play an important role in gut homeostasis and limiting intestinal inflammation [16-18].

Given the dual regulatory effect of MSCs, we hypothesized that MSCs, which modulate immune cells including Treg cells, may have effective anti-inflammation effects on colitis and eventually suppress CAC. To test this hypothesis, MSCs were obtained and injected intravenously in CAC mouse. The therapeutic effects of MSCs on both inflammation and tumor stage of CAC were investigated.

## Methods

### Mice and CAC model

The CAC model was induced in C57BL/6 male mice (eight weeks of age) purchased from the Model Animal Research Center of Nanjing University. All of the animals received care according to the Guide for the Care and Use of Laboratory Animals. The protocol was approved by the Committee on the Ethics of Animal Experiments of Nanjing University Medical School. Mice were divided into four groups: normal group untreated with MSCs (n = 12); normal group treated with MSCs (n = 12); tumor group untreated with MSCs (n = 16); and tumor group treated with MSCs (n = 16). After treatment with intraperitoneal azoxymethane (AOM) (10 mg/kg, 13.4 M, purity ≥98%; SIGMA, Aldrich, St. Louis MO, USA), three cycles of 2% (w/v) DSS (40,000 Da; SIGMA, Aldrich, St. Louis MO, USA) in the drinking water (7 days DSS and 14 days water) induced long-lasting chronic DSS colitis in these mice, as previously described [19]. Mice were checked daily for behavior. At day 5 and day 26, mice were intravenously injected with 2 × 10^5^ MSCs diluted in 200 μl phosphate-buffered saline (PBS) or a vehicle control (PBS alone) through the tail vein. The mortality rate of this animal model is 0% to 5%. Half of the mice in each group were sacrificed at day 33 to investigate the progression of inflammation, and the others were sacrificed at day 70 to evaluate the development of tumor. The degree of colitis was evaluated daily by scoring the clinical disease activity (0 to 4), which includes stool status, fecal blood and weight loss. Colons removed from cecum to anus were collected, photos were taken, and the colons prepared for histopathological analysis, immunohistochemistry and quantitative real-time PCR (Q-PCR) analysis.

### Preparation of umbilical cord-derived MSCs

Human umbilical cords were obtained from the Affiliated Drum Tower Hospital of Nanjing University Medical School. The Drum Tower Hospital ethics committee approved the consent forms and the protocol for evaluation of the tissue and patients gave written consent. The umbilical cords were rinsed with PBS in penicillin and streptomycin, and then the umbilical arteries and veins were removed. The remaining tissue was cut into 1 to 2 mm pieces and floated in (Dulbecco’s) modified Eagle’s medium/nutrient F-12 Ham’s (Gibco, Grand Island, NY, USA). The pieces were subsequently digested in an enzyme cocktail (hyaluronidase 5U/ml, collagenase 125U/ml, and dispase 50U/ml; SIGMA) for three hours at 37°C. After this tissue was crushed with forceps and large pieces were removed, human umbilical cord MSCs were harvested and plated into a culture flask. The cells were incubated at 37°C in an incubator with 5% CO_2_ at saturating humidity. When cells developed colonies and reached 70% to 80% confluence, the cells were detached with 0.25% trypsin-ethylenediaminetetraacetic acid (EDTA) and passaged to a culture flask for further expansion.

### Determination of MSC migration

MSCs migrating to the intestines *in vivo* were detected by labeling with the fluorescent dye CM-DiI (Molecular Probes Eugene, Oregon, USA). Before being injected, MSCs were resuspended in PBS containing 2 μg CM-DiI, and then incubated at 37°C for five minutes followed by fifteen minutes at 4°C in the dark. Cells were washed twice with PBS and resuspended to be injected in the tail vein. Mice were killed (at day 8 and day 12) and tissues were collected to make paraffin sections. MSCs were determined by fluorescence microscope detection of CM-DiI dye (red).

### Histopathological analysis

For histopathological examination, intestinal tissue was embedded in paraffin, sectioned and stained with hematoxylin and eosin (H & E). Inflammation was blindly scored from 0 to 4 as follows: 0, no signs of inflammation; 1, low leukocyte infiltration; 2, moderate leukocyte infiltration; 3, high leukocyte infiltration, moderate fibrosis, high vascular density, thickening of the colon wall, moderate goblet cell loss, and focal loss of crypts; and 4, transmural infiltration, massive loss of goblet cells, extensive fibrosis, and diffuse loss of crypts.

### Immunohistochemistry

Paraffin-embedded intestinal sections were mounted on slides, dewaxed in xylene, and rehydrated in graded alcohol washes. Slides were heated by microwave in 0.01 mol/L tri-sodium citrate buffer for antigen retrieval. Bovine serum albumin, 5%, (BSA) was used to block nonspecific bonding sites for 30 minutes and 3% H_2_O_2_ was used to suppress endogenous peroxidase activity. Slides were then incubated with primary antibody (Ki-67, p-P65 and FoxP3) overnight at 4°C and washed with Tris-buffered saline (TBS) before incubation with labeled polymer horseradish peroxidase rabbit antibody for 30 minutes. Counterstaining was performed with hematoxylin. Slides were dehydrated through ascending alcohols to xylene and mounted to take photos. The proliferation index of Ki-67 and p-P65 was given as the ratio between positive nuclei and total number of nuclei per crypt. Five visual fields per sample were selected randomly to analyze at least 20 crypts.

### mRNA isolation and quantitative-PCR

Total RNA was extracted from intestinal tissue with TRIzol Reagent (Invitrogen, Carlsbad, CA, USA) according to the manufacturer’s instructions. A total of 1 μg RNA was used as the template for single strand cDNA synthesis. Quantitative PCR (Q-PCR) for GAPDH, IL-1β, IL-6 and TNF-α was performed on an Applied Biosystems 7300 Sequence Detection System (Applied Biosystems, Foster city CA, USA) using SYBR green dye (Invitrogen, USA), programmed for 95°C for 10 minutes, then 40 cycles of 95°C for 15 seconds, 60°C for 30 seconds, and 72°C for 30 seconds. All reactions were run in triplicate.

### Cytokine analysis by Bio-Plex Assay

IL-α, IL-β, IL-3, IL-4, IL-5, IL-6, IL-12, IL-13, G-CSF and RANTES in sera were quantitated to investigate the change in the immune system using the 10-plex Bio-Rad (Bio-Rad, Hercules CA, USA), according to the manufacturer’s instructions.

### T cell preparation and treatment

CD4^+^CD62L^+^ T cells were positively selected from murine splenocytes using a magnetic-activated cell-sorting system (Miltenyi Biotec, Bergisch Gladbach Germany). High purity naive T cells were plated into 12-well culture plates and cultured in Roswell Park Memorial Institute (RPMI) 1640 (Gibco, Grand Island, NY, USA) containing 10% fetal bovine serum (FBS) and ant-CD3/CD28 (eBioscience, San Diego, CA, USA) monoclonal antibodies. Then MSCs were added in the top chamber of 12-well transwell chambers with 3-μm pore polycarbonate filters and co-cultured with naive T cells at 37°C in an incubator with 5% CO_2_ at saturating humidity. Transforming growth factor-β1 (TGF-β1) (Miltenyi Biotec Bergisch Gladbach, Germany) and anti-TGF-β1 antibody (ab64715, Abcam, USA) was also added in the same chamber to evaluate the effect of MSCs on the differentiation of naive T cells. Overall, we had four groups: CD4^+^T cells alone, CD4^+^T cells cultured with TGF-β, CD4^+^T cells cultured with MSCs, and CD4^+^T cells cultured with anti-TGF-β/MSCs. Cells at the bottom of the chamber were collected and analyzed.

### Flow cytometric analysis

Cells were stained with CD29, CD44, CD73, CD90, CD105, HLA-ABC, CD14, CD31, CD34, CD45, HLA-DR, CD3, CD4, CD8, CD25, IL-4, IL-17, and FoxP3 (eBioscience). In addition, intracellular IL-4, IL-17 and FoxP3 staining were treated with a FoxP3 staining buffer set (eBioscience). Flow cytometry was carried out on the FACScalibur flow cytometer (BD, San Diego, USA). Data were analyzed using FlowJo software (Treestar, Inc., San Carlos, CA, USA).

### Western blots

Jurkat cells, which were stimulated by MSCs conditioned medium, were lysed in a buffer containing 50 mM Tris-Cl pH 8.0, 150 mM NaCl, 0.02% NaN3, 0.1% SDS, 100 mg/ml phenyl-methylsufonyl fluoride (PMSF), 1 mg/ml aprotinin, 1% Triton. A total of 10 μg/mL aprotinin, 10 μg/mL leupeptin, 1 mM dithiothreitol, 1 mM paranitrophenyl phosphate, and 0.1 mM Na_3_VO_4_ were added as protease and phosphatase inhibitors. After centrifugation, protein samples were subjected to 10% SDS-PAGE and transferred onto polyvinylidene difluoride (PVDF) membranes (Roche, Mannheim, Germany). The membranes were blocked in TBST (1 mM Tris–HCl, pH 7.4, 150 mM NaCl, 0.05% Tween-20) containing 5% BSA for 0.5 hour and subsequently incubated overnight at 4°C with diluted primary antibodies against GAPDH, Smad2 and Phospho-Smad2. The band was quantified using the FluorChem FC2 system (Alpha Innotech Corporation St. Leonardo, CA, USA).

### Statistical analysis

Results are presented as mean ± standard error of the mean (SEM). Student’s t-test was used to compare between two groups. One-way analysis of variance (ANOVA) was used to compare among three or more groups. A *P* value of <0.05 was considered significant, and survival curves were performed with GraphPad Prism software (San Diego, CA, USA).

## Results

### MSCs migrate to the colon and decrease the incidence of colitis-related neoplasm

**Figure 1:**
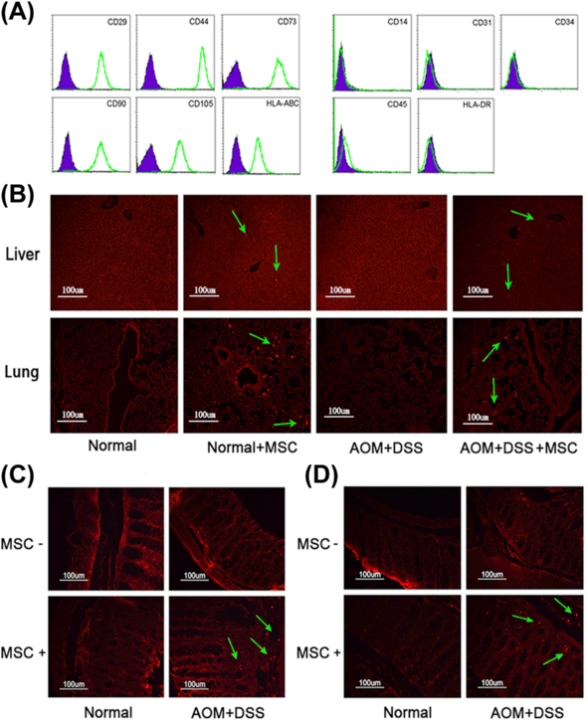
The phenotype and migration pathway of MSCs in the DSS/AOM-induced model. **(A)** Expression of MSC phenotype was detected by FACScalibur flow cytometer. **(B)** Liver and lung in each group were moved, collected and paraffin sections made at day 6 to detect the migration pathway of MSCs stained with CM-DiI (red). **(C)** and **(D)** show colon tissue collected to detect the migration pathway of MSCs at day 6 and day 10. AOM, azomethane; DSS, dextran sulfate sodium; FACS, fluorescence activated cell sorting; MSCs, mesenchymal stem cells.

MSCs were isolated from human umbilical cord tissue and confirmed by analyzing MSC-related cell surface antigens using flow cytometry. As shown in Figure 1A, MSCs were successfully obtained with positive markers of CD29, CD44, CD73, CD90, CD105 and HLA-ABC, and with negative markers of CD14, CD31, CD34, CD45 and HLA-DR. We then detected the migration activity of MSCs in mice through tail vein injection. Compared with liver, more accumulation of MSCs was found in lung for three days after injection in normal mice. In the tumor group, that is, the mice treated with AOM and 2% DSS, the amount of MSCs in lung was decreased (Figure 1B). At the same time, MSCs were also detected in colon (Figure 1C). These results indicated that MSCs were sufficiently dynamic to migrate *in vivo* and could migrate to colon tissues when the intestinal epithelia of mice were damaged by DSS. At day 12, MSCs still were found in the colon tissue (Figure 1D), suggesting that this time interval provided a potential therapeutic chance of MSCs in colon tissues.

**Figure 2:**
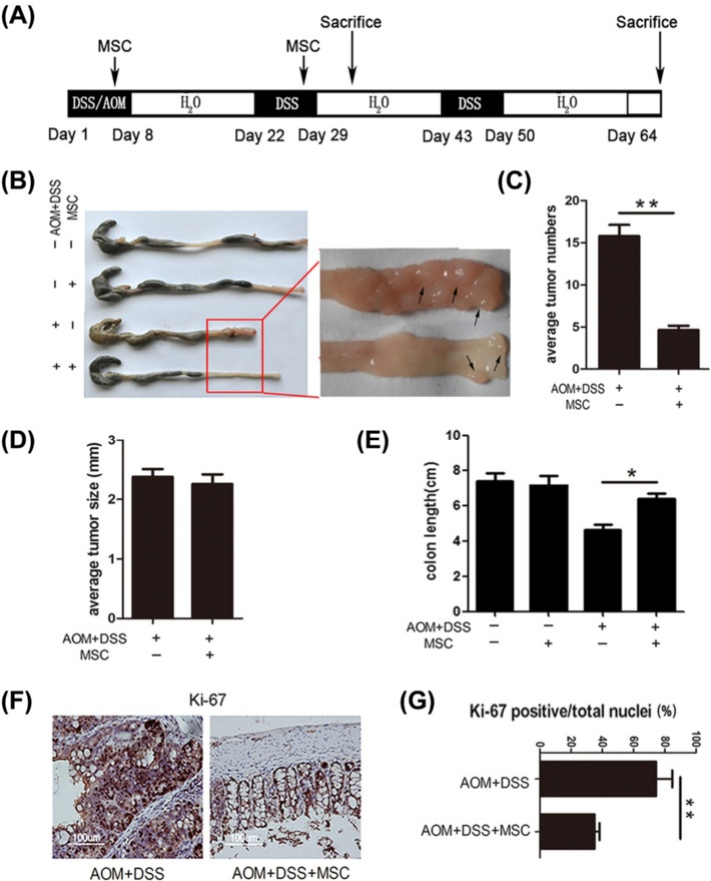
MSCs decrease tumor incidence on day 70. **(A)** After AOM injection, animals were subjected to three cycles of DSS. MSCs (2 × 10^5^) were injected at day 4 and day 25. Animals were sacrificed at day 33 and day 70. Colons were excised for macroscopic observation **(B)** and tumor numbers **(C)**, tumor size **(D)** and colon length were assessed **(E)**. The expression of Ki-67 **(F** and **G)** in the treated and untreated colons was examined by immunohistochemistry staining. Values are expressed as means ± SEM. *P <0.05, or **P <0.01. AOM, azomethane; DSS, dextran sulfate sodium; MSCs, mesenchymal stem cells; SEM, standard error of the mean.

The CAC model was successfully induced by injecting mice with AOM and 2% DSS (Figure 2A). Mice in the tumor group developed colon tumors mainly in the distal to middle colon, which is the predominant localization of human colorectal tumors. We noticed that mice treated with MSCs had fewer macroscopic tumors (Figure 2B and C) and longer colon length (Figures 2E). The average size of the tumor was not affected in the two groups (Figure 2D). Moreover, immunohistochemical analysis showed that Ki-67 was lower in colon tissues of mice treated with MSCs than in those without treatment (Figure 2F and G). These data confirmed that MSCs could inhibit tumor development in the CAC model.

### MSCs alleviate the pathology of DSS-induced chronic colitis

**Figure 3:**
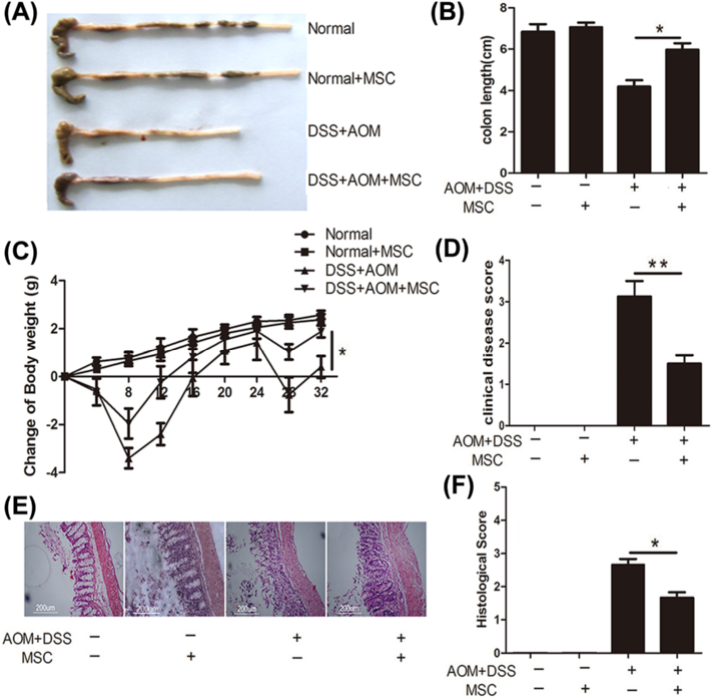
MSCs protect mice against the development of DSS-induced colitis on day 33. Colons were excised for macroscopic observation **(A)** and assessment of colonic length **(B). (C)** The changes in body weight were calculated based on initial body weight. **(D)** The clinical disease score of colitis, which includes stool status, fecal blood, length of colon and weight loss, was used to evaluate the therapeutic effect of MSCs. **(E)** Colon sections from mice with different treatments were examined using H & E staining. **(F)** Histology scores were derived from microscopic analysis of longitudinal colon sections from each mouse. Values are expressed as means ± SEM. *P <0.05, or **P <0.01. DSS, dextran sulfate sodium; MSCs mesenchymal stem cells; SEM, standard error of the mean.

To induce colorectal cancer successfully, the chronic colitis should emerge first in three cycle inductions. It is now commonly accepted that inflammation contributes to the initiation, promotion, and progression of tumor development. To confirm that MSCs inhibit carcinogenesis through inhibition of colonic inflammation, half of the mice in the tumor group were sacrificed on day 32. As shown in Figures 3A to D, MSC treatment significantly alleviated chronic inflammation with improved clinical scores. MSC treatment also improved colitis characteristics with increased body weight (*P* <0.05) and colon length (*P* <0.05). Moreover, histological examination of the colon in mice without MSCs showed patchy ulceration, epithelial cell loss, reduction of the density of tubular glands, focal loss of crypts, inflammatory cell infiltrates and transmural inflammation involving all layers of the bowel wall. Expectedly, these colitis characteristics were reversed after MSC treatment (Figures 3E and F, *P* <0.05). These results consistently demonstrated that during the development of CAC, MSCs had a protective function on DSS-induced colitis by inhibiting excessive inflammation in colon tissues.

### MSCs inhibit inflammatory cytokines in colon and serum

**Figure 4:**
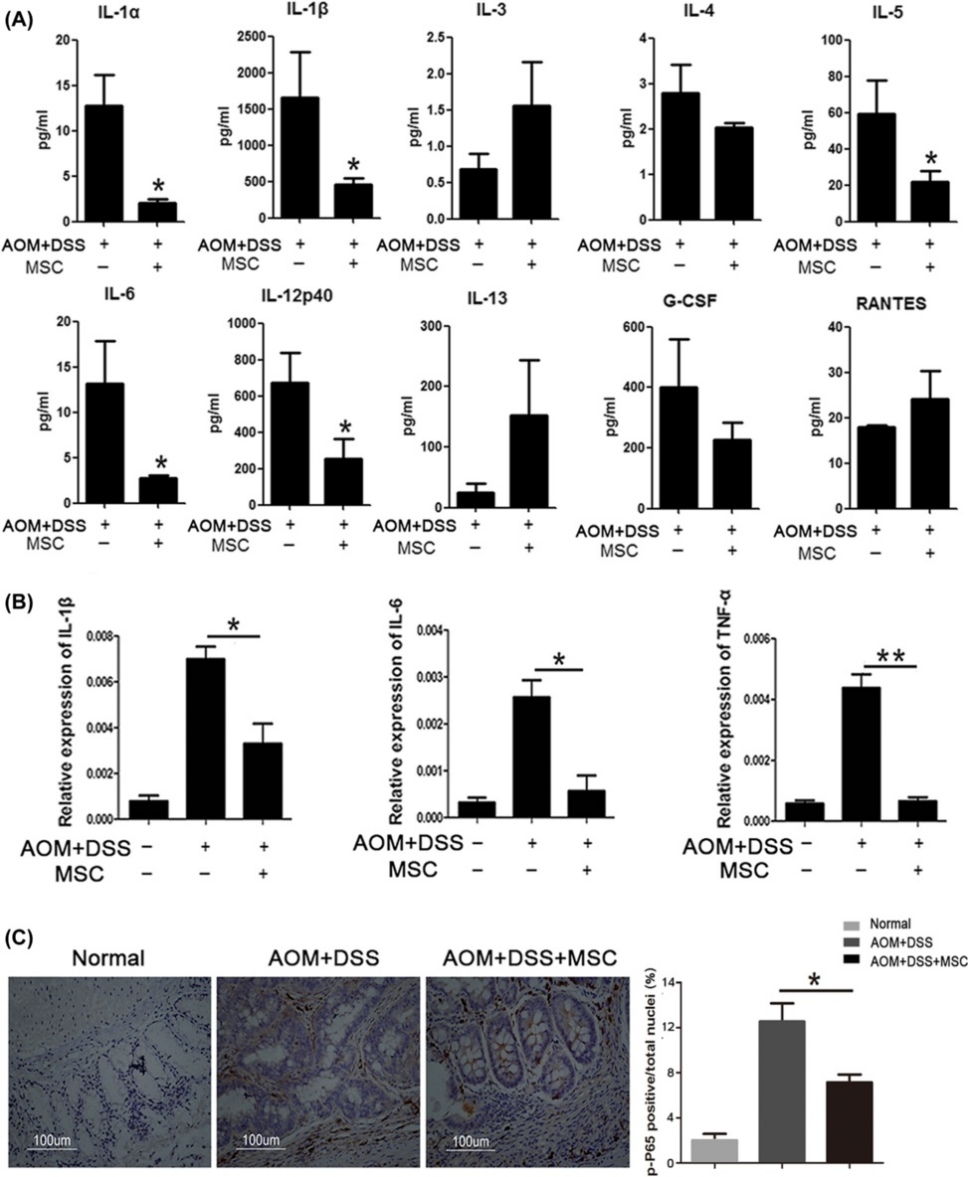
MSCs suppressed the expression of inflammatory cytokines. **(A)** Bio-Plex Assay was used to analyze cytokine changes in mice plasma, including IL-1α, IL-1β, IL-3, IL-4, IL-5, IL-6, IL-12, IL-13, G-CSF and RANTES. **(B)** The expression levels of colonic inflammatory cytokines (IL-1β, IL-6 and TNF-α) from different groups were evaluated by Q-PCR. **(C)** The expression of p-P65 examined by immunohistochemistry staining comparing the colons in the treated and untreated groups. Values are expressed as means ± SEM. *P <0.05, or **P <0.01. MSCs, mesenchymal stem cells; SEM, standard error of the mean.

Cytokine levels are critical evidence to show the effect of immune cells on CAC growth. In order to test the effect of MSCs on levels of inflammatory cytokines, serum from different groups of mice was collected and cytokine levels were detected using a Bio-Plex cytokine kit. Compared with the untreated group, inflammatory cytokines, including IL-1α, IL-1β, IL-5, IL-6 and IL-12, were decreased significantly after treatment with MSCs (Figure 4A, *P* <0.05).

Many DNA-binding proteins are aberrantly activated in response to inflammatory stimuli, which can cause inappropriate induction of various proinflammatory genes in tumor cells, tumor-associated stromal cells and surrounding host tissues. NF-κB, one of these DNA-binding proteins, has been identified as a potential molecular bridge between inflammation and cancer, and can induce proinflammatory cytokines such as IL-1, IL-6 and TNF-α. mRNA in colon was extracted and gene expressions of IL-1β, IL-6 and TNF-α were examined. As shown in Figure 4B, the expressions of IL-1β, IL-6 and TNF-α were reduced (*P* <0.05) after treatment with MSCs. Immunohistochemical analysis also showed that the protein level of p-P65 was decreased in colon tissues treated with MSCs, compared with colon tissues without treatment (Figure 4C).

### Treg cells are up-regulated in the progression of chronic colitis

**Figure 5:**
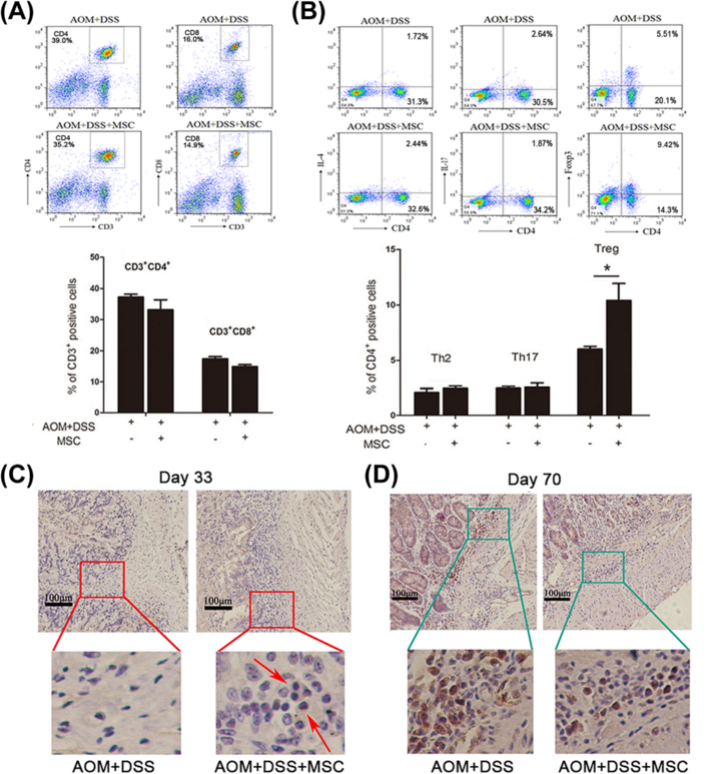
The effect of MSCs on the accumulation of CD4^+^CD25^+^FoxP3^+^Treg cells. **(A)** Flow cytometry analysis of the mean percentage of CD4^+^T cells and CD8^+^T cells in mesenteric lymph. **(B)** Flow cytometry analyzed the mean percentage of Th2, Th17 and Treg cells in mesenteric lymph. Values are expressed as means ± SEM. *P <0.05. Colons collected at day 33 **(C)** and day 70 **(D)** stained with Foxp3 examined by immunohistochemistry. MSCs, mesenchymal stem cells; SEM, standard error of the mean; Treg cells, regulatory T cells.

To detect the regulatory effect of MSCs on immune cells, flow cytometry was used to analyze changes of adaptive immune cells in mesenteric lymph nodes after treatment with MSCs for 33 days (colitis stage). As shown in Figure 5A, the mean percentage of CD4^+^T cells and CD8^+^T cells reflected no difference among groups as well as Th2 and Th17 cells. As is well known, forkhead box P3 (FoxP3) is the transcription factor of Treg cells. Notably, CD4^+^CD25^+^FoxP3^+^ Treg cells were significantly up-regulated by MSC treatment (Figure 5B, *P* < 0.05). These phenomena suggest that MSC may just induce the accumulation of Treg cells in mesenteric lymph nodes to suppress excessive inflammation after MSCs migrate to the colon. To further confirm the involvement of Treg cells in intestinal inflammation, FoxP3 stained by immunohistochemistry was used to analyze whether Treg cells infiltrated to the stroma and epithelia of the intestine. Interestingly, according to the density of FoxP3, we found that Treg cells infiltrated in intestinal stroma more in the MSC treated group than in the untreated group (Figure 5C). We also determined the density of Treg cells at day 70 (tumor stage). In contrast, the results showed that the infiltrated Treg cells were found less in the MSCs group compared to the neoplasm in the untreated group (Figure 5D). Therefore, these results indicated that MSCs indeed were involved in inducing Treg cells to suppress colitis.

### MSCs increase Foxp3+ cells via activating Smad2 signaling *in vitro*

**Figure 6:**
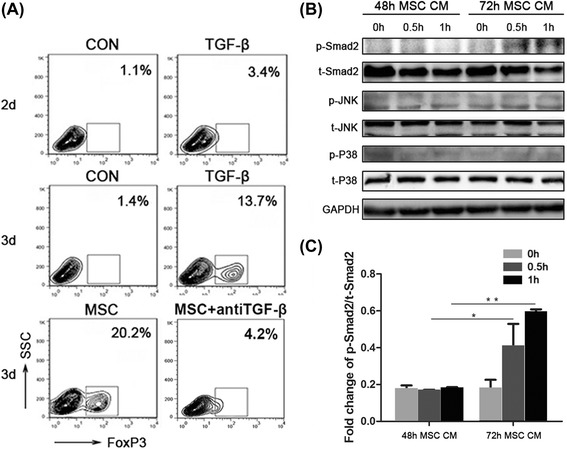
MSCs increase the expression of FoxP3 in naive CD4^+^T cells. **(A)** Naive CD4^+^T cells co-cultured with MSCs (ratio 1:5)/TGF-β (5 ng/ml) or without MSCs/TGF-β in the presence of anti-CD3/CD28 (2 μg/ml each). T cells were harvested from different treatment groups to assess Foxp3 expression by flow cytometry. **(B)** MSCs conditioned medium was collected after 48 hours and 72 hours, and then used to stimulate Jurkat cells for different times. were harvested to examine TGF-β signaling by Western blotting of Smad2 and phospho-Smad2. TCR signaling was examined by Western blotting of JNK, phospho-JNK, p38 and phospho-p38. **(C)** The protein level of p-Smad2 was analyzed using Quantity One. Values are expressed as means ± SEM. *P <0.05 or **P <0.01. MSCs, mesenchymal stem cells; SEM, standard error of the mean; TCR, T cell receptor; TGF-β, transforming growth factor-β.

To explore how MSCs regulate Treg cells, naive CD4^+^T cells were separated from mouse spleen and then stimulated by anti-CD3/CD28 to maintain survival *in vitro*. After co-culture with MSCs for two days, the expression of FoxP3 slightly changed (Figure 6A). However, after three days, the FoxP3^+^ cells were increased to 13.7% in the TGF-β alone group and 20.2% in the MSC group. To prove that the induction effect of MSCs is through TGF-β, anti-TGF-β was added to the MSC supernatant. The percentage of FoxP3^+^ cells returned to its normal level in the anti-TGF-β group. Moreover, the MSC conditioned medium was then used to stimulate Jurkat cells. Mechanically, phosphorylation-Smad2 was activated after 30 minutes treatment with MSCs while JNK and p38 signaling showed no change (Figure 6B and C). These data suggest that MSCs secreting TGF-β increased the FoxP3^+^ cell populations via activating phosphorylation of Smad2.

## Discussion

As it is difficult to cure tumors directly, many reports have focused on anti-inflammation to inhibit the initiation and progression of tumors [20]. Recently, some studies have indicated that paeonol [20], glucosamine [21], and digitoflavone [22] can significantly attenuate inflammation and produce proinflammatory cytokines to ameliorate CAC. Our previous research showed that MSCs can regulate immunological balance [23-25] and ameliorate DSS-induced acute colitis [9]. Based on these studies, we designed this experiment to research whether MSCs may inhibit tumorigenesis in the CAC mice model. Our results found that MSCs could migrate to the colon and suppress colitis-related neoplasms. This tumor suppressive effect was characteristized by longer colon length, decreased tumor numbers and decreased expression of Ki-67. Furthermore, MSCs alleviated the pathology of inflammation in the colitis stage of the CAC model. Mechanically, Treg cells were accumulated in mesenteric lymph node of MSC-treated mice. *In vitro*, MSCs secreting TGF-β enhanced the induction of Treg cells from naïve T cells.

The effect of MSCs on cancers is controversial. Many studies have shown that MSCs can promote tumor growth. B16 melanoma cells cannot form tumors in allogeneic mice unless co-injected with MSCs [26]. When human breast cancer cells mixed with bone marrow-derived human MSCs were injected in a mouse model, there was a higher speed of metastases [27]. On the other hand, there are also many reports proving that MSCs can inhibit tumor growth. MSCs can exert an anti-tumorigenic effect that is mediated by inhibition of Akt activity in a model of Kaposi’s sarcoma [28]. MSCs can also inhibit human liver cancer cell lines and the development of a hepatoma model involving the wnt signaling pathway [29]. However, there is much debate about the function of MSCs in tumor development, and it is difficult to research how MSCs suppress tumors directly. In our CAC model, we found that MSCs could suppress colitis at the colitis stage and eventually suppress tumor development at the tumor stage. Furthermore, inflammatory mediators were detected both in blood and intestinal tissues. IL-1β, IL-6 and TGF-β, which had high levels in colitis [30], were significantly decreased in the MSC treated group. These results together suggest that MSCs can play a therapeutic role in the progression of CAC.

It is believed that Treg cells play an important role in the regulation of colitis. Colitis induced by transferring CD4^+^CD25RB^high^ T cells into RAG^−/−^ mice can be prevented by CD4^+^CD25^+^ Treg cells [31]. Modulating the balance between Treg and Th17 cells by IL-22 can also reduce the pathological degree in experimental colitis [32]. Here, we found that at the colitis stage, both the colon and mesenteric lymph node had more infiltration of Treg cells in MSC treated group than in the untreated group. On the other hand, our results found that at the tumor stage fewer Treg cells were infiltrated in the MSCs treated group compared with the untreated group. Here, we just injected MSCs twice to suppress inflammation development at the colitis stage, so at the tumor stage whether Treg cell infiltration could be affected by MSCs needs further research.

FoxP3, which has been identified as the transcription factor, is responsible for determining the function of Treg cells. Various signals that induce the expression of FOXP3 have been reported, such as T-cell receptor (TCR), co-stimulatory molecules and cytokine receptors [33]. IL-2 [34] and TGF-β [35] are essential cytokines for the differentiation of CD4 + CD25 + FoxP3 + Treg cells from naive CD4 + T cells. It has been reported that MSCs can secrete TGF-β to interact with NK cells [36]. *In vitro*, we found that Treg cells were induced when naive CD4 + T cells were co-cultured with MSCs and then the TGF-β-Smad2 signal was also activated. All these results support our hypothesis that MSCs may secrete TGF-β which was quantified in our previous study [24] to induce Treg cells, and then suppress the progression of inflammation-dysplasia-carcinoma in CAC.

## Conclusions

To sum up, our present research analyzed the function of MSCs in CAC. We found that MSCs can inhibit colitis at the colitis stage and eventually suppress the development of colon cancer at the tumor stage. The expressions of inflammatory cytokines in blood and colon tissue were all inhibited by MSCs. Importantly, Treg cells accumulated in colon tissues following MSC treatment. MSCs can raise the expression of FoxP3 in naive CD4 + T cells and activate the TGF-β-Smad2 signal. Taken together, our results prove that MSCs can induce Treg cells to suppress chronic inflammation and then suppress the development of CAC.

## Abbreviations

AOM: azoxymethane
BSA: bovine serum albumin
CAC: colitis-associated colorectal cancer
DSS: dextran sulfate sodium
FoxP3: forkhead box P3
hUCMSCs: human umbilical cord tissue-derived mesenchymal stem cells
IFN: interferon
IL: interleukin
PBS: phosphate-buffered saline
Q-PCR: quantitative PCR
TGF-β: transforming growth factor-β
TNF: tumor necrosis factor
Treg cells: regulatory T cells
